# Early intraocular pressure kinetics after intravitreal aflibercept 8 mg (0.07 mL) versus aflibercept 2 mg (0.05 mL) in nonglaucomatous eyes: a prospective comparative study

**DOI:** 10.1186/s40942-026-00906-2

**Published:** 2026-08-19

**Authors:** Annika Licht, Astrid Schneider, V. Romanou, Alireza Mirshahi

**Affiliations:** 1 Dardenne Eye Hospital, Bonn, Germany; 2 Independent Statistician, Mainz, Germany

**Keywords:** Intraocular pressure, Intravitreal injection, Aflibercept, Reference model, Ocular dynamics

## Abstract

**Background:**

Transient intraocular pressure (IOP) spikes after intravitreal injection (IVI) are expected. The higher-volume aflibercept 8 mg (0.07 mL) formulation has recently entered clinical use, yet its early IOP kinetics—particularly the immediate post-injection trajectory in eyes without known glaucoma or ocular hypertension—remain incompletely characterised.

**Methods:**

In this prospective, non-randomised, single-centre comparative study, 48 eyes of 48 patients received intravitreal aflibercept 8 mg/0.07 mL (*n* = 25) or aflibercept 2 mg/0.05 mL (*n* = 23). Eyes with any history of glaucoma or ocular hypertension were excluded. IOP was measured at baseline and at 3, 10, and 30 min after injection using iCare IC200 tonometry. The primary objective was to assess within-group IOP changes from baseline in the 0.07-mL group. A hierarchical fixed-sequence procedure was applied using paired Wilcoxon signed-rank tests with Hodges–Lehmann (HL) estimates and 95% confidence intervals (CIs). Between-group differences were explored using a linear mixed-effects model adjusted for baseline IOP, age, sex, and lens status.

**Results:**

Baseline characteristics were comparable between groups. In the 0.07-mL group, HL pseudo-median IOP changes from baseline were + 25.5 mmHg (95% CI 18.5 to 32.0; *p* < 0.001) at 3 min, + 11.0 mmHg (95% CI 8.0 to 15.0; *p* < 0.001) at 10 min, and + 3.5 mmHg (95% CI 1.5 to 5.5; *p* = 0.002) at 30 min. Adjusted between-group differences (0.07 mL minus 0.05 mL) were + 5.8 mmHg (95% CI 0.3 to 11.3; *p* = 0.038) at 3 min, + 2.8 mmHg (95% CI − 2.7 to 8.3; *p* = 0.31) at 10 min, and + 0.2 mmHg (95% CI − 5.3 to 5.7; *p* = 0.94) at 30 min. Two eyes in the 0.07-mL group received topical IOP-lowering medication after the 30-minute assessment; no paracentesis was performed.

**Conclusions:**

In eyes without known outflow disease, intravitreal aflibercept 8 mg (0.07 mL) was associated with a higher early IOP spike than aflibercept 2 mg (0.05 mL). However, the pressure increase was transient, and exploratory between-group analyses suggested convergence of IOP levels by 30 min. By defining the early IOP trajectory in eyes with intact outflow, these data provide a physiological reference for the short-term ocular safety of higher-volume anti-VEGF therapy and a benchmark against which exaggerated or delayed responses in at-risk eyes can be interpreted.

**Trial registration:**

Not applicable (observational study).

## Background

Short-term intraocular pressure (IOP) elevations after anti-VEGF IVI are common and largely related to the injected volume, scleral/ocular rigidity and outflow capacity. Meta-analytic evidence confirms an acute rise that typically dissipates within 24 h and no consistent long-term IOP increase with anti-VEGF therapy (de Vries, 2020). The recently introduced aflibercept 8 mg formulation delivers a larger intravitreal bolus (0.07 mL versus 0.05 mL), raising the question of whether this additional volume produces a clinically meaningful difference in the immediate IOP response. Early comparative reports have begun to address this question, but most have pooled glaucomatous and non-glaucomatous eyes, did not include the earliest post-injection timepoint at which the pressure peak occurs, or sampled IOP at only one or two timepoints after injection. As a result, the physiological short-term IOP response to the higher injection volume, isolated from outflow-related confounding, has not been clearly defined. Nevertheless, a time-resolved, physiological reference curve in non-glaucomatous eyes—particularly for aflibercept 8 mg/0.07 mL versus 2 mg/0.05 mL—has not been established. We therefore prospectively characterised the complete early IOP regression curve at 3, 10, and 30 min post-injection under standardised conditions in a deliberately non-glaucomatous cohort, to provide a normative baseline against which at-risk populations can be compared.

## Methods

### Study design and ethics

Prospective, non-randomised, single-centre comparative study conducted at the Dardenne Eye Hospital (Bonn, Germany) from March to September 2025. The protocol adhered to the Declaration of Helsinki and was approved by the institutional ethics committee (Northrhine chamber of physicians, ID 2025004). Written informed consent was obtained from all participants.

### Participants

Adults (≥ 18 years) receiving aflibercept for neovascular AMD or diabetic macular oedema were eligible. Exclusion criteria were any history of glaucoma or OHT (> 24 mmHg), prior uveitis, prior pressure-lowering procedures (selective laser trabeculoplasty, MIGS, iridotomy/iridectomy, filtering surgery, paracentesis) or intraocular surgery within six months as well as prescription of IOP-lowering medication in the last six months. This restriction was chosen to capture physiological IOP regression without outflow-related confounding. One eye per patient was included.

## Injection protocol

All injections were performed by the same retinal specialist (VR) using a 30-gauge needle (BD Microlance ™ 3, Becton Dickinson S.A.) and pars plana entry 3.5 mm posterior to the temporal inferior limbus. Injection duration was not measured; however, no bolus injections were performed. All injections were administered slowly and in a controlled manner. Study medications were aflibercept 8 mg/0.07 mL or aflibercept 2 mg/0.05 mL in pre-filled syringes.

Two eyes (one in each group) with observable subconjunctival reflux were excluded from further measurements to avoid reflux-mediated attenuation of IOP peaks. No prophylactic IOP-lowering therapy was used.

### IOP measurement

IOP was measured with iCare^®^ IC200 at T0 (baseline, pre- IVI), T1 (3 min), T2 (10 min) and T3 (30 min). Two readings per timepoint were taken and averaged. Measurements were performed in the sitting position under consistent ambient conditions. Immediately after the needle was withdrawn, hand motion was checked to see if the optic nerve head was perfused. The study endpoint was IOP measured at T0, T1, T2, and T3.

### Statistical analysis

Sample size planning was based on a paired t-test and used the mean within-subject change in IOP between the preoperative measurement and the 10-minute postoperative measurement reported in a previous study (+ 4.6 mmHg with an standard deviation (SD) of the paired differences of 7.0 mmHg) [12]. Assuming a two-sided significance level of α = 0.05 and 80% power, the required sample size was *n* = 20.18, corresponding to 21 participants after rounding. Due to a subsequent deviation from normal distribution in the collected study data, the non-parametric Wilcoxon signed-rank test was ultimately applied instead of the paired t-test.

Continuous variables were summarised using mean ± SD and median (interquartile range [IQR]), as well as minimum and maximum values. Categorical variables were reported as absolute and relative frequencies; this included the proportions of patients reaching specific clinical thresholds, such as IOP ≥ 40 or ≥ 30 mmHg at any time point, and an increase of ≥ 3 mmHg above baseline at T3. Temporal IOP changes were visualised for both groups using boxplots. IOP Change measured from baseline of + 3 mmHg or higher was rated as clinically relevant.

The primary objective was to assess within-group IOP changes from T0 to T1, T2, and T3 in the 0.07mL group. To strictly control the type I error rate, these changes were evaluated via a hierarchical fixed-sequence procedure. Within-group comparisons were performed using paired Wilcoxon signed-rank tests with Hodges–Lehmann (HL) estimates and 95% CIs. Statistical significance was defined as *p* < 0.05. P-values are reported with three decimal places for values < 0.05 and two decimal places for values ≥ 0.05. For values below 0.001, *p* < 0.001 is reported.

Secondary objectives included within-group changes in the 0.05mL group and between-group contrasts at each timepoint. The latter were analysed via a linear mixed-effects model (random intercept for subject) adjusted for baseline IOP, age, sex, and lens status. Between-group and 0.05mL group analyses were considered exploratory; thus, p-values for these comparisons are reported without further adjustment for multiplicity.

## Results

### Baseline characteristics

Baseline demographics and clinical characteristics are summarized in Table 1. Patients were stratified by injection volume (0.07 mL vs. 0.05 mL aflibercept). Age and sex distribution was comparable between groups. Baseline IOP was slightly higher in the 0.07 mL group; however, this difference was not statistically significant (*p* = 0.67, Wilcoxon rank-sum test). After injection, IOP was higher at all timepoints in the 0.07 mL group (Table 1).

**Table 1:** Baseline characteristics: Baseline characteristics and IOP before and after intravitreal injection (IVI) in the overall study population (*N* = 48) and stratified by injection volume: 0,07 mL aflibercept (*N* = 25) and 0,05 mL aflibercept (*N* = 23). Sex and lens status are presented as n(%), and minimum- maximum. Intraocular pressure IOP timepoints: T0 = pre-IVI, T1 = 3 min post-IVI, T2 = 10 min post-IVI, T3 = 30 min post-IVI. Abbreviations: IOP; IVI = intravitreal injection; SD = standard deviation; Q1/Q3 = first/third quartile

	Study population *N* = 48	0.07 mL aflibercept *N* = 25	0.05 mL aflibercept *N* = 23
**Gender, female**	28 (58%)	12 (48%)	16 (70%)
**Age (in years)**			
Mean, SD Median/Q1-Q3 Min - Max	77/11 78/70–85 38 – 95	77/11 77/68–85 53–95	77/12 80/ 72–85 38–91
**Lens status**,** pseudophakic**	31 (65%)	15 (60%)	16 (70%)
**IOP pre IVI (T0)**			
Mean/SD Median/Q1-Q3 Min – Max	13/4 13/ 10–16 8–22	14/ 4 14/ 10–16 8–22	13/ 3 12/ 11–16 8–20
**IOP 3 min post IVI (T1)**			
Mean, SD Median/Q1-Q3 Min – Max	35/ 16 35/ 26–47 7–68	38 / 17 37/ 27–51 7–68	31 / 14 30/ 24–39 7–61
**IOP 10 min post IVI (T2)**			
Mean/std Median/Q1-Q3 Min – Max	23/ 11 23/ 16–28 5–56	25/11 24/17–31 6–56	20/ 9 19/ 12–27 5–36
**IOP 30 min post IVI (T3)**			
Mean/std Median/Q1-Q3 Min – Max	16/ 6 15/ 11–20 7–35	17/7 16/12–20 7–35	15/ 5 14/ 11–20 9–23

### Primary outcome: Intra-group IOP changes over time in 0.07mL group

**Fig. 1:**
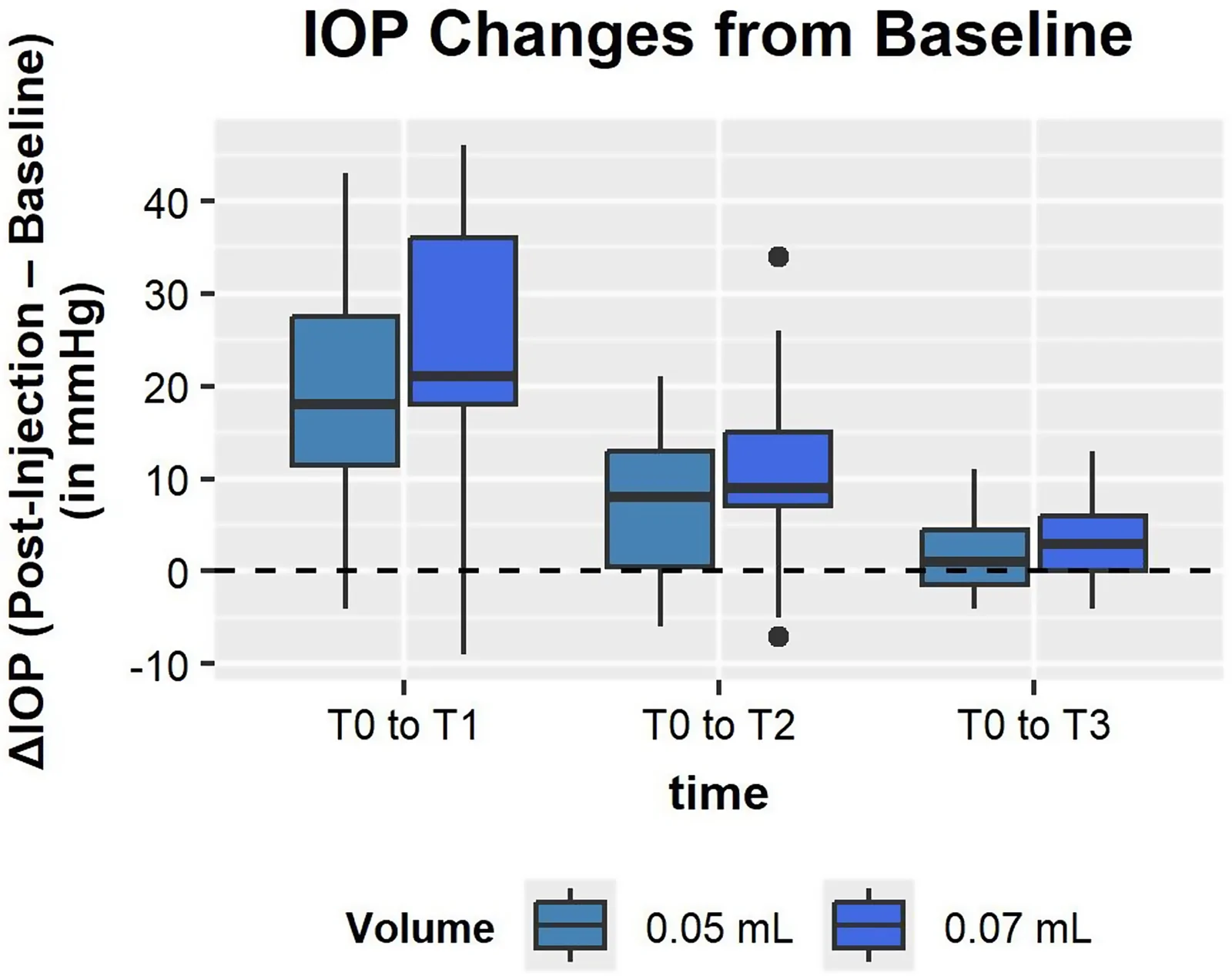
IOP changes from Baseline in 0.07mL and 0.05mL Aflibercept treatment group

As shown in Fig. 1, both groups exhibited a pronounced increase in IOP shortly after injection. The median IOP change from T0 to T1 was higher in the 0.07 mL group than in the 0.05 mL group (median differences reported in Fig. 1).

All within-group comparisons for the 0.07 mL group reached statistical significance within the hierarchical fixed-sequence testing procedure. In this group, IOP increased significantly from baseline at all time points according to Wilcoxon signed rank tests: the Hodges–Lehmann (HL) estimated change was + 25.5 mmHg (95% CI 18.5–32.0; *p* < 0.001) at 3 min, + 11.0 mmHg (95% CI 8.0–15.0; *p* < 0.001) at 10 min, and + 3.5 mmHg (95% CI 1.5–5.5; *p* = 0.002) at 30 min.

### Secondary outcome: intra-group IOP changes over time in 0.05mL group and between-group comparisons

In the 0.05 mL group, within-group analyses were considered exploratory. Similar to the 0.07 mL group, nominal increases in IOP from baseline were observed at all time points, although the magnitude of change was lower. According to Wilcoxon signed rank tests: the HL estimated increases were + 19.0 mmHg (95% CI 14.0–24.0; nominal *p* < 0.001) at 3 min, + 8.5 mmHg (95% CI 4.5–12.0; nominal *p* = 0.001) at 10 min, and + 2.0 mmHg (95% CI 0.0–4.5; nominal *p* = 0.042) at 30 min.

At T1, IOP ≥ 40 mmHg occurred in 48% (*N* = 12, 0.07 mL) vs. 22% (*N* = 5, 0.05 mL). 2 Patients (007–9, 007–25) still had high IOP (> 30mmHg) at T3 30 min after IVI with 0.07mL aflibercept and required local therapy (1 drop brimonidine + 1 drop dorzolamide + timolol). 1 h after IVI, both patients reached eye pressure < 25mmHg without paracentesis.

The linear mixed-effects model revealed that adjusted mean IOP differed between the two groups immediately following injection. At 3 min (T1), the 0.07 mL group exhibited a higher IOP compared to the 0.05 mL group (adjusted mean difference: +5.8 mmHg; 95% CI 0.3–11.3; nominal *p* = 0.038). This difference diminished over time, with no clear disparities observed at 10 min (+ 2.8 mmHg; 95% CI − 2.7 to 8.3; *p* = 0.31) or 30 min (+ 0.198 mmHg; 95% CI − 5.3 to 5.7; *p* = 0.94).

**Table 2:** Between-group comparison and sensitivity analysis of IOP recovery: Primary Model adjusted for age, sex, lens status and baseline IOP T0, Sensitivity Model adjusted for baseline IOP T0, other variables did not show significant effect (*p* > 0.05)

Analysis Model	Parameter/Comparison	Difference (95% CI)	*p*-value
Primary Model	Between-Group Differences (0.07mL – 0.05mL)		
	T1 3 min	+ 5.80 [0.32, 11.29]	0.038
	T2 10 min	+ 2.81 [-2.67, 8.29]	0.31
	T3 30 min	+ 0.20 [-5.29, 5.68]	0.94
	Interaction (Group x Time (T2 vs. T1)*	-2.99 [-7.89, 1.90]	0.24
	Interaction (Group x Time (T3 vs. T1)*	-5.60 [-10.50, -0.71]	0.029
Sensitivity Model	T1 (3 min)	+ 6.14 [0.77, 11.50]	0.026
	T2 (10 min)	+ 3.14 [-22, 8.51]	0.25
	T3 (30 min)	+ 0.53 [-4.84, 5.90]	0.84
	Interaction (Group x Time (T2 vs. T1)*	-2.99 [-7.89, 1.90]	0.24
	Interaction (Group x Time (T3 vs. T1)*	-5.60 [-10.50, -0.71]	0.029

* For all comparisons and interaction effects, Group 0.05mL serves as the reference group, and Time T1 (3 min) serves as the reference time point

T0 was identified as a key determinant of post-injection IOP levels. A substantial positive association was observed (beta = 1.52, 95% CI [0.89, 2.15], p < 0.001), indicating that higher initial pressures levels were associated with consistently higher absolute IOP values. To maintain model parsimony and account for the sample size, T0 was modelled as a common fixed effect across all time points, as the data did not support the inclusion of a “T0 x time” interaction.”

To explore IOP stability 30 min after treatment (T3), we determined the proportion of patients whose IOP levels were at most 3 mmHg above their baseline values (T0). This increase was defined as clinically tolerable; IOP values falling below the baseline were also considered within this range of stability.

In the 0.05 mL group (*n* = 23), 65% (*n* = 15) of patients reached this defined stability range, while 60% (*n* = 15) did so in the 0.07 mL group (*n* = 25). A comparison using Fisher’s Exact Test yielded a p-value of 0.77. Additionally, a notable IOP decrease (more than 3 mmHg below baseline) was observed in 4% (*n* = 1) of patients in the 0.05 mL group and in 8% (*n* = 2) of patients in the 0.07 mL group.

**Fig. 2:**
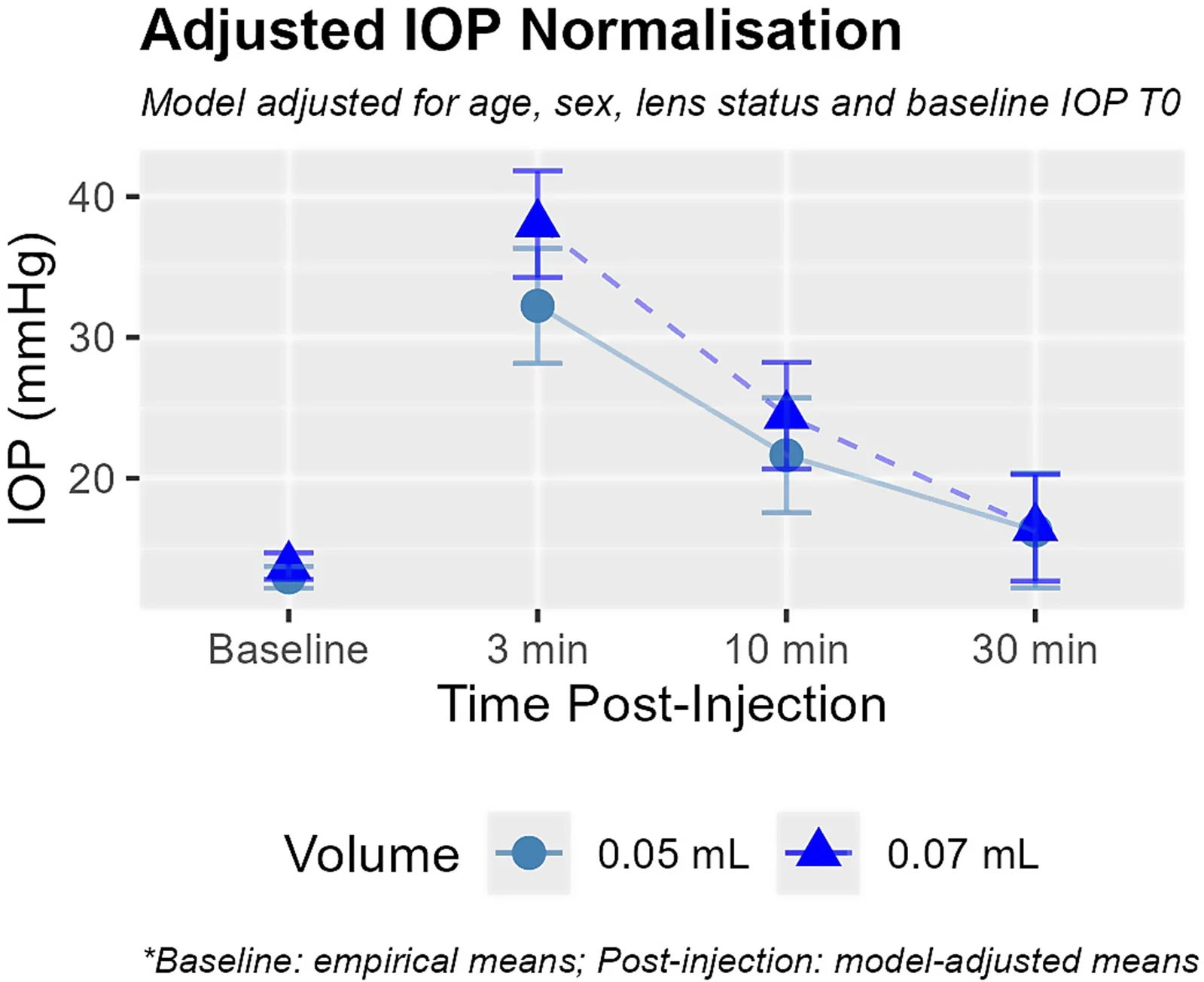
Adjusted IOP Normalisation for 0.07 mL and 0.05mL after IVI in mmHg

A notable interaction between group and time was observed regarding the rate of IOP reduction. The decrease in IOP from T1 to T3 was 5.6 mmHg greater in the 0.07 mL group than in the 0.05 mL group (nominal *p* = 0.029), reflecting a steeper recovery curve from the higher initial peak. Baseline covariates, including age (*p* = 0.73), sex (*p* = 0.76), and lens status (*p* = 0.32), were not associated with IOP levels. The full adjusted model can be seen in Table 2. Figure 2 illustrates the model-adjusted IOP normalisation over time for each volume group, controlled for baseline IOP, age, sex, and lens status. The raw IOP values for both groups at each timepoint are summarised in Table 1. A sensitivity analysis using a reduced model (including only baseline IOP T0, group, and time) yielded similar effect estimates and is shown in Table 2.

## Discussion

This prospective comparative study describes the early IOP profile after intravitreal aflibercept 8 mg (0.07 mL) in eyes without known glaucoma or ocular hypertension. Both treatment groups showed a pronounced early IOP increase followed by rapid decline. The 0.07-mL group demonstrated a greater IOP rise at 3 min than the 0.05-mL group, whereas adjusted between-group differences were no longer evident at 30 min. Within the 0.07-mL group, IOP remained modestly but significantly above baseline at 30 min, indicating that pressure normalisation may be incomplete within the first half hour in some eyes. The immediate rise and rapid regression we observed is consistent with prior reports for 0.05 mL injections [6, 12]Compared with earlier reports using 0.05 mL injections, the main difference was the magnitude of the immediate IOP spike. The larger bolus delivered with aflibercept 8 mg resulted in a markedly greater pressure increase at 3 min; however, this early separation diminished rapidly, and IOP no longer differed significantly between groups at 30 min.The residual elevation above baseline observed at 30 min in the 0.07 mL group, although small, reached the prespecified threshold for clinical relevance, whereas the corresponding change in the 0.05 mL group remained below this threshold.Its principal implication is that full normalisation may occasionally extend beyond the first half hour, especially after receiving higher injection volumes.

The short-term IOP increase we observed (+ 25–30 mmHg) fits within the range summarized by de Vries et al., [2] in their meta-analysis of 74 studies [2]. Our data complement those findings by providing a time-resolved physiological curve, a benchmark that can be used to identify abnormal responses in eyes with compromised outflow.

Recent research identified glaucoma as the strongest predictor of sustained IOP elevation 30 min after intravitreal injection [11]. This underscores the importance of analysing a cohort without pre-existing IOP-related pathology to characterise physiological recovery. Unlike prior investigations that mixed glaucomatous and non-glaucomatous eyes [4, 8, 11] or did not assess immediate post-injection IOP measurement at 3 min [5, 9, 10], the present study deliberately excluded eyes with impaired outflow to isolate normal IOP dynamics following higher-volume intravitreal injections. To our knowledge, this is the first prospective study to define a detailed early IOP regression curve—from the immediate 3-minute peak through to 30 min—for the higher-volume aflibercept 8 mg in a cohort free of outflow disease. Consequently, our dataset serves as a physiological baseline against which delayed or exaggerated post-injection IOP responses can be interpreted. Compared with slower recovery in mixed or glaucomatous populations [8], this underscores the importance of intact outflow for pressure regulation.

In our cohort, the only two eyes that had not normalised by 30 min both belonged to the 0.07 mL group, and both shared a single distinguishing feature: a borderline-elevated baseline IOP. These two eyes (both from male patients) also produced the highest immediate peaks recorded in the study. Their behaviour suggests that, even without diagnosed glaucoma, a relatively high baseline IOP may flag the small subset of eyes in which the larger injection volume yields a more pronounced and slower-resolving pressure response. In both cases, topical IOP-lowering drops (brimonidine and Cosopt^®^ (dorzolamide/timolol)) were given after the 30-minute measurement, reducing IOP to 23 mmHg at 45 min (patient 007–25) and to 24 mmHg at 90 min (patient 007–9).

In addition, at 3 min post IVI (T1), IOP values ≥ 40 mmHg were more than twice as common in the 0.07 mL group (48%, 12/25) as in the 0.05 mL group (22%, 5/23). Spikes above 50 mmHg may compromise optic nerve perfusion and have been reported in up to one third of standard-volume (0.05 mL) bevacizumab injections [7]. While such IOP peaks are usually well tolerated in eyes with healthy optic nerves, they may be non-trivial in patients with advanced glaucoma. Prophylactic treatment with topical brimonidine mitigates this early spike, supporting a mechanical explanation [3]. In our cohort, an IOP spike > 50 mmHg occurred in 4% of eyes in the 0.05 mL group (1/23) compared with 28% in the 0.07 mL group (7/25; mean 57.43 mmHg, median 54 mmHg, SD ± 6.72 mmHg).

An important consideration when interpreting these findings is the upper measurement limit of the iCare IC200. At T1, 7 of 25 eyes (28%) in the 0.07 mL group recorded IOP values above 50 mmHg, exceeding the device’s specified measurement range. Consequently, the true peak IOP in these eyes could not be accurately quantified and may have been underestimated. This limitation is particularly relevant because the primary endpoint of the study was the IOP change at 3 min after injection. Any underestimation of values above 50 mmHg would be expected to bias the estimated T1 IOP increase toward lower values. Therefore, the observed HL pseudo-median increase at T1 should be interpreted as a conservative estimate, and the true difference between the 0.05 mL and 0.07 mL injection groups may be greater than reported. However, because the magnitude of underestimation above the device’s measurement ceiling is unknown, the extent of this potential bias cannot be quantified reliably.

### Methodological strengths

We prespecified a hierarchical confirmatory framework centered on T1; reported HL pseudo-medians with CIs for paired changes; and used an adjusted LMM with group×time to describe decay dynamics. Multiple early timepoints (3/10/30 min) capture the trajectory, in contrast to two-timepoint designs that risk misclassification.

### Technical and anatomical modifiers

Subconjunctival reflux blunts peaks; we excluded such eyes to avoid bias. Needle gauge and injection speed may also influence peak magnitude. Smaller injection needles were found to have a higher IOP increase, possibly because the bigger injection needles leave a greater opening and therewith allow more reflux than smaller needles [6]. Age-related scleral stiffening could modulate ΔP/ΔV as previous research suggests [1]; but our small sample showed no age effect on 30-min regression.

### Implications for clinical practice

For non-glaucomatous eyes, post-IVI IOP spikes are mostly self-limiting and rarely require intervention. Eyes receiving the 0.07 mL volume with a baseline IOP ≥ 21 mmHg, even in the absence of diagnosed glaucoma, may benefit from pre-injection IOP-lowering measures such as topical IOP-lowering drops.

Clinically, our results help define typical early IOP responses after IVI in non-glaucomatous eyes, with convergence of IOP values between the two injection volumes by 30 min. These data can serve as control parameters for studies evaluating prophylactic strategies or long-term glaucomatous risk [2, 8].

### Limitations

This study has several limitations. First, the non-randomised design limits causal inference and does not fully exclude selection bias. Second, eyes with visible subconjunctival reflux were excluded, and the frequency of reflux was not recorded, which may limit generalisability. Third, follow-up ended at 30 min, so later IOP normalisation could not be assessed. Fourth, because the iCare IC200 has a specified measurement range of 7–50 mmHg, extreme post-injection values above 50 mmHg (iCare-world.de, technical data IC200) https://www.icare-world.com/product/icare-ic200-tonometer/, checked 11.06.26, 7 pm) should be interpreted as approximate rather than equally precise point estimates. Injection speed was also not standardised and represents a potential source of unmeasured within-study variability. Finally, the modest sample size limited statistical power for subgroup analyses and adjustment for additional confounders.

## Conclusions

In summary, intravitreal aflibercept 8 mg (0.07 mL) produced a greater early IOP spike than aflibercept 2 mg (0.05 mL) in eyes without known glaucoma or ocular hypertension. This increase was transient, and adjusted between-group differences were no longer significant at 30 min. The data suggest that most non-glaucomatous eyes tolerate the higher injection volume without invasive intervention, although delayed recovery may occur in individual eyes, particularly when baseline IOP is relatively high. By describing the early IOP trajectory in eyes without pre-existing IOP-related pathology, these findings provide a physiological reference that may inform pre-injection risk assessment and guide future studies of prophylactic strategies and of higher-risk populations.

## Data Availability

No datasets were generated or analysed during the current study.

## Abbreviations

Afl: aflibercept
Anti-VEGF: Anti-vascular endothelial growth factor
DME: Diabetic macular oedema
IOP: Intraocular pressure
IVI: Intravitreal injection
LMM: Linear mixed-effects model
nAMD: Neovascular age-related macular degeneration
OHT: Ocular hypertension
